# The development of a nomogram and the prognostic prediction value of patients with esophageal squamous cell carcinoma undergoing radical radiotherapy

**DOI:** 10.2144/fsoa-2021-0080

**Published:** 2022-01-24

**Authors:** Ling Xiao, Jiahua Lyu, Meihua Chen, Liu Wang, Jing Huang, Yajun Wei, Tao Li

**Affiliations:** 1 School of Medicine, University of Electronic Science & Technology of China, Chengdu, China; 2 Department of Radiotherapy Centre, Sichuan Cancer Hospital & Institute, Chengdu, China; 3 Affiliated Hospital of Chengdu University of Traditional Chinese Medicine (West District), Chengdu Pidu District Hospital of Traditional Chinese Medicine

**Keywords:** chemoradiotherapy, ESCC, prediction model

## Abstract

**Aim:**

This study aimed to explore the role of the developed nomogram in the prognosis of esophageal squamous cell carcinoma (ESCC).

**Methods:**

A total of 181 ESCC patients were randomly divided into a training cohort (n = 141) and a validation cohort (n = 40). Significant factors impacting overall survival (OS) were identified in the training set and integrated into the nomogram based on Cox proportional hazards regression.

**Results:**

In the training cohort, the median OS in the high group (≥222) was 49.9 months and the median OS in the low group (<222) was 14.4 months. Multivariate analysis revealed that age, Karnofsky performance status score, tumor stage, chemotherapy, BMI, cervical esophageal carcinoma index and neutrophil to lymphocyte ratio were predictors of OS.

**Conclusion:**

The developed nomogram can effectively predict the survival prognosis of ESCC patients.

## Lay abstract

Currently, the prognosis of patients with esophageal squamous cell carcinoma (ESCC) is poor, especially in patients with advanced stages. This study aimed to establish a prognostic model based on the common detection indexes and treatment status of different ESCC patients during treatment. Studies have shown that the prognosis of ESCC patients is related to local tumor size, nutritional function and immune inflammatory indicators. The prognostic model established by us can better distinguish patient risk and predict patients’ overall survival. Pre-treatment intervention for patients with a poor prognosis may improve the survival of ESCC patients.

In recent years, esophageal cancer has become one of the main causes of malignant tumor morbidity and mortality worldwide [1]. In China the main pathological type is squamous cell carcinoma [2]. Although progress has been made in the combined treatment of surgery, radiotherapy (RT) and chemotherapy, the prognosis of patients with esophageal squamous cell carcinoma (ESCC) remains poor due to early recurrence or distant metastasis. Previous studies have shown that the prognosis of ESCC patients is affected by smoking history, drinking history, regional eating habits and treatment [3–5]. In addition, research shows that blood-related indicators before treatment can be used to establish predictive models; these indicators include the platelet to lymphocyte ratio (PLR), prognostic nutrition index (PNI), lymphocyte to monocyte ratio, systemic immunity inflammation index (SII) and neutrophil to lymphocyte ratio (NLR), as these factors are significantly correlated with the prognosis of ESCC patients [6–10]. However, there are certain differences in the various reports on these models. Therefore we suggest establishing a scoring system based on PLR, PNI, SII, NLR and other models.

The new prediction model could be used to build a multivariate regression model and convert complex regression equations into visual graphs, making the prediction model results more intuitive and easier to evaluate. Few studies have used laboratory indicators to establish ESCC prognostic models. Therefore this study collected clinical data and laboratory test indicators of ESCC patients and established prognostic factors and scoring models to help clinicians more accurately determine the prognosis of ESCC patients. This model can potentially be used to ensure timely intervention and improve the prognosis of ESCC patients.

## Patients & methods

### Patients

We collected the data from ESCC patients who underwent radical chemoradiotherapy or RT at Sichuan Cancer Hospital from 20 March 2012 to 25 December 2017. A total of 181 patients were finally included. Patients were randomly divided into a training cohort (141 patients) and a verification cohort (40 patients). All patients included in the analysis met the following inclusion criteria: ESCC confirmed by histology or cytology; patients refused or could not tolerate surgery; Karnofsky performance status (KPS) score ≥70 points; radiation therapy dose ≥50 Gy (in 25–35 fractions over a range of 5–7 weeks) and between zero and six courses of chemotherapy at the same time; data obtained through blood biochemical examinations 1 week before treatment; no distant tumor metastasis; and reclassified according to the tumor node metastasis (TNM) classification system issued by the American Joint Committee on Cancer (7th edition).

### Treatments

All patients received radical RT with or without chemotherapy. The RT dose was 50–72.6 Gy. The primary tumor and positive regional lymph nodes were defined as the gross tumor volume. A radial edge of 0.5–1.0 cm was provided around the gross tumor volume, and the proximal and distal 3-cm edges were defined as the clinical target volume. Tumor movement was 0.5 cm of clinical target volume expansion to define the planned target volume. Patients who received concurrent chemotherapy were mainly administered platinum monotherapy or combination chemotherapy.

### Definition of indicators

At 1 week before treatment, the total number of lymphocytes in blood cells and serum albumin levels as an indication of liver function were collected (PNI = serum albumin level [g/l] + 5 × absolute lymphocyte count). NLR is the ratio of the absolute number of neutrophils to the absolute number of lymphocytes. PLR is the ratio of the platelet count to absolute lymphocyte count. SII is defined as the platelet count multiplied by the NLR. The cervical esophageal carcinoma index (CEI) is obtained by multiplying the BMI by the serum albumin concentration (g/l)/NLR [7]. The optimal cutoff values for SII, PNI, NLR, PLR, CEI and SII were calculated using the maximum value of the Youden index.

### Follow up

From the time the patient was admitted to the hospital, all patients were followed up through outpatient examinations and telephone calls. Detailed information regarding the patient’s basic situation was collected, the results were reviewed and the survival status was determined. The last follow up was on 15 January 2020. The survival time was measured from the first day of pathological diagnosis to death or the last follow up. The median follow-up time was 21.6 months (range: 2–91 months).

### Statistical analysis

SPSS version 25.0 (IBM Corp., NY, USA) and GraphPad Prism version 8.0.2 (GraphPad, CA, USA) were used to analyze all recorded data. The *t*-test or analysis of variance was used to compare continuous variables with a normal distribution. The χ^2^ test was used to compare count data. The Kaplan–Meier method was used to calculate the overall survival (OS) rate. Univariate and multivariate analyses were conducted using Cox proportional hazard models to study the impact of different factors on survival. p < 0.05 was considered to indicate a statistically significant difference. The nomogram model was constructed using the survival and rms packages of R3.6.3 software. The factors with p < 0.05 in the Cox multifactor analysis were included, and the study end points were 1-year and 3-year OS rates. The total score of each patient was calculated and patients were divided into a low group and high group according to the best cutoff value. The accuracy of the model prediction was evaluated using a consistency index (concordance index, C-index) and calibration curves. Receiver operating characteristic (ROC) curves were used to assess the prognostic model and each prognostic factor curve (area under the curve [AUC]).

## Results

### Basic clinical data

This study screened patients strictly according to the inclusion and exclusion criteria and finally included 181 patients, including 123 males (67.96% of the total cases) and 58 females (32.04% of the total cases). The age range was 41–86 years, with an average age of 63 years. The most common tumor sites were the middle thoracic segment (42.54%), followed by the upper thoracic segment (38.67%), cervical segment (11.05%) and lower thoracic segment (7.73%). Covariates included age (<67 vs ≥67 years), gender, smoking history, drinking history, KPS score, tumor location, tumor length (<5 vs ≥5 cm), clinical tumor (T) stage, clinical node (N) stage, TNM stage, RT dose (<66 vs ≥66 Gy), chemotherapy, BMI (<21.13 vs ≥21.13), PNI (<45.95 vs ≥45.95), PLR (<217.2 vs ≥217.2), SII (<1497 vs ≥1497), CEI (<1729 vs ≥1729) and NLR (<4.84 vs ≥4.84). Table 1 shows the comparison of patient factors in the training and verification cohorts.

**Table 1.** Distribution of various factors in the training and verification cohorts.

Characteristic	Patients, n (%)	Training cohort (n = 141)	Validation cohort (n = 40)	p-value
Age (years)				0.316
≤66	110 (60.77)	83	27	
>66	71 (39.23)	58	13	
Sex				0.249
Male	123 (67.96)	111	12	
Female	58 (32.04)	30	28	
Smoking history				0.634
No	80 (44.2)	61	19	
Yes	101 (55.8)	80	21	
Drinking history				0.868
No	79 (43.65)	62	17	
Yes	102 (56.35)	79	23	
KPS score				0.357
70, 80	97 (53.59)	73	24	
90	84 (46.41)	68	16	
Localization				0.161
Cervical	20 (11.05)	16	4	
Upper thoracic	70 (38.67)	59	11	
Middle thoracic	77 (42.54)	56	21	
Lower thoracic	14 (7.73)	10	4	
Tumor length (cm)				0.280
<5.0	77 (42.54)	57	20	
≥5.0	104 (57.46)	84	20	
T stage				0.005
T2	13 (7.18)	6	7	
T3	79 (43.65)	60	19	
T4	89 (49.17)	75	14	
N stage				0.553
N0	1 (0.55)	0	1	
N1	67 (37.02)	53	14	
N2	83 (45.86)	63	20	
N3	30 (16.57)	25	5	
TNM stage				0.052
II	8 (4.42)	4	4	
III	173 (95.58)	137	36	
RT dose (Gy)				0.652
<66	58 (32.04)	44	14	
≥66	123 (67.96)	97	26	
Chemotherapy				0.622
No	23 (12.71)	17	6	
Yes	158 (87.29)	124	34	
BMI (kg/m^2^)				0.231
<21.13	80 (44.2)	59	21	
≥21.13	101 (55.8)	82	19	
PNI				0.255
<45.95	44 (24.31)	37	7	
≥45.95	137 (75.69)	104	33	
PLR				0.352
<217.2	134 (74.03)	121	13	
≥217.2	47 (25.97)	20	27	
SII				0.838
<1497	166 (91.71)	129	37	
≥1497	15 (8.29)	12	3	
CEI				0.631
<1729	46 (25.41)	37	9	
≥1729	135 (74.59)	104	31	
NLR				0.175
<4.84	150 (82.87)	114	36	
≥4.84	31 (17.13)	27	4	

CEI: Cervical esophageal carcinoma index; KPS: Karnofsky performance status; N: Node; NLR: Neutrophil to lymphocyte ratio; PLR: Platelet to lymphocyte ratio; PNI: Prognostic nutritional index; RT: Radiotherapy; SII: Systemic immunity inflammation index; T: Tumor; TNM: Tumor node metastasis.

### Univariate & multivariate survival analysis

In the training cohort, the data from 141 patients were subjected to Cox regression analysis to identify the factors influencing prognosis. In the univariate analysis, age, KPS, tumor length, T stage, N stage, TNM stage, chemotherapy, BMI, PNI, PLR, SII, CEI and NLR showed a significant impact on the prognosis of ESCC patients (p < 0.2) [11]. The factor analysis results showed that age (hazard ratio [HR]: 1.990; 95% CI: 1.288–3.074; p = 0.002), KPS score (HR: 0.509; 95% CI: 0.330–0.785; p = 0.002), T stage (HR: 5.838; 95% CI: 1.628–20.929; p = 0.007), chemotherapy (HR: 0.471; 95% CI: 0.254–0.875; p = 0.017), BMI (HR: 0.574; 95% CI: 0.376–0.878; p = 0.010), CEI (HR: 0.489; 95% CI: 0.293–0.819; p = 0.006) and NLR (HR: 4.447; 95% CI: 2.555–7.739; p < 0.001) were independent factors affecting the prognosis. In addition, age, T stage and NLR were identified as risk factors, whereas KPS, BMI, CEI and chemotherapy were protective factors (Table 2).

**Table 2.** Univariate and multivariate analysis of prognostic factors for overall survival in the training cohort.

Characteristic	Univariate analysis		Multivariate analysis	
	HR (95% CI)	p-value	HR (95% CI)	p-value
Age (years)				
<67	1		1	
≥67	2.044 (1.356–3.080)	0.001	1.990 (1.288–3.074)	0.002
Sex				
Female	1		1	
Male	0.775 (0.474–1.267)	0.310	–	–
Smoking history				
No	1		1	
Yes	1.044 (0.694–1.571)	0.835	–	–
Drinking history				
No	1		1	
Yes	1.269 (0.841–1.915)	0.257	–	–
KPS score				
70–80	1		1	
90	0.660 (0.436–0.997)	0.048	0.509 (0.330–0.785)	0.002
Localization				
Cervical	1		1	
Upper thoracic	1.212 (0.476–3.087)	0.687	–	–
Middle thoracic	0.881 (0.394–1.969)	0.757	–	–
Lower thoracic	0.847 (0.377–1.906)	0.689	–	–
Tumor length (cm)				
<5.0	1		1	
≥5.0	1.448 (0.953–2.202)	0.083	1.326 (0.822–2.136)	0.247
T stage				
T2	1		1	
T3	1.937 (0.596–6.292)	0.271	5.838 (1.628–20.929)	0.007
T4	2.879 (0.894–9.268)	0.076	7.672 (2.222–26.498)	0.001
N stage				
N1	1		1	
N2	0.968 (0.615–1.523)	0.888	0.952 (0.564–1.606)	0.853
N3	1.584 (0.914–2.742)	0.101	1.313 (0.68–2.535)	0.418
TNM stage				
II	1		1	
III	2.661 (0.653–10.853)	0.172	2.768 (0.219–34.897)	0.431
RT dose (Gy)				
<66	1		1	
≥66	0.991 (0.642–1.530)	0.969	–	–
Chemotherapy				
No	1		1	
Yes	0.425 (0.244–0.741)	0.003	0.471 (0.254–0.875)	0.017
BMI (kg/m^2^)				
<21.13	1		1	
≥21.13	0.482 (0.319–0.727)	0.001	0.574 (0.376–0.878)	0.010
PNI				
<45.95	1		1	
≥45.95	0.567 (0.364–0.883)	0.012	0.989 (0.549–1.783)	0.971
PLR				
<217.2	1		1	
≥217.2	2.357 (1.381–4.022)	0.002	1.339 (0.606–2.96)	0.471
SII				
<1497	1		1	
≥1497	3.904 (2.040–7.470)	<0.001	1.221 (0.467–3.191)	0.684
CEI				
<1729	1		1	
≥1729	0.673 (0.432–1.047)	0.079	0.489 (0.293–0.819)	0.006
NLR				
<4.84	1		1	
≥4.84	2.384 (1.486–3.825)	<0.001	4.447 (2.555–7.739)	< 0.001

CEI: Cervical esophageal carcinoma index; HR: Hazard ratio; KPS: Karnofsky performance status; N: Node; NLR: Neutrophil to lymphocyte ratio; PLR: Platelet to lymphocyte ratio; PNI: Prognostic nutritional index; RT: Radiotherapy; SII: Systemic immunity inflammation index; T: Tumor; TNM: Tumor node metastasis.

### Nomogram model

**Figure 1.**
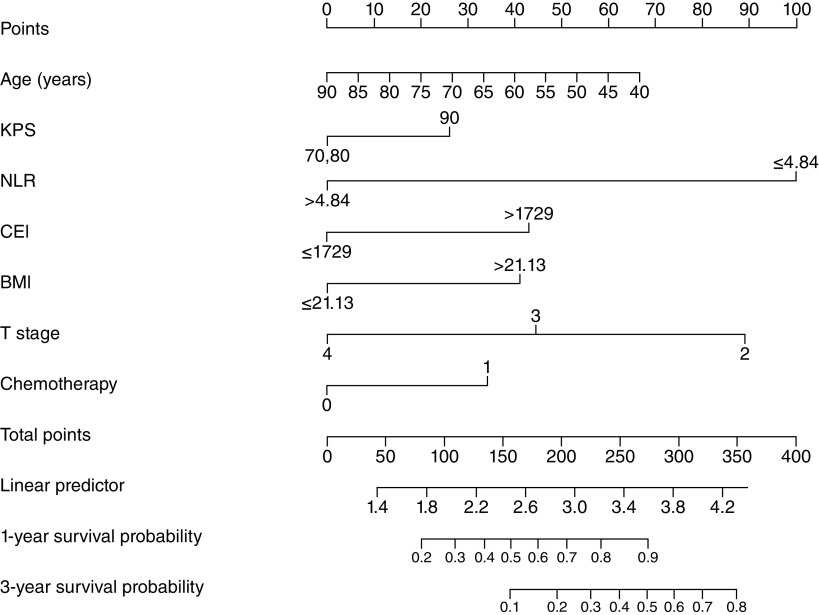
Nomogram for predicting 1-year and 3-year overall survival of esophageal squamous cell carcinoma. CEI: Cervical esophageal carcinoma index; KPS: Karnofsky performance status; NLR: Neutrophil to lymphocyte ratio; T: Tumor.

**Figure 2.**
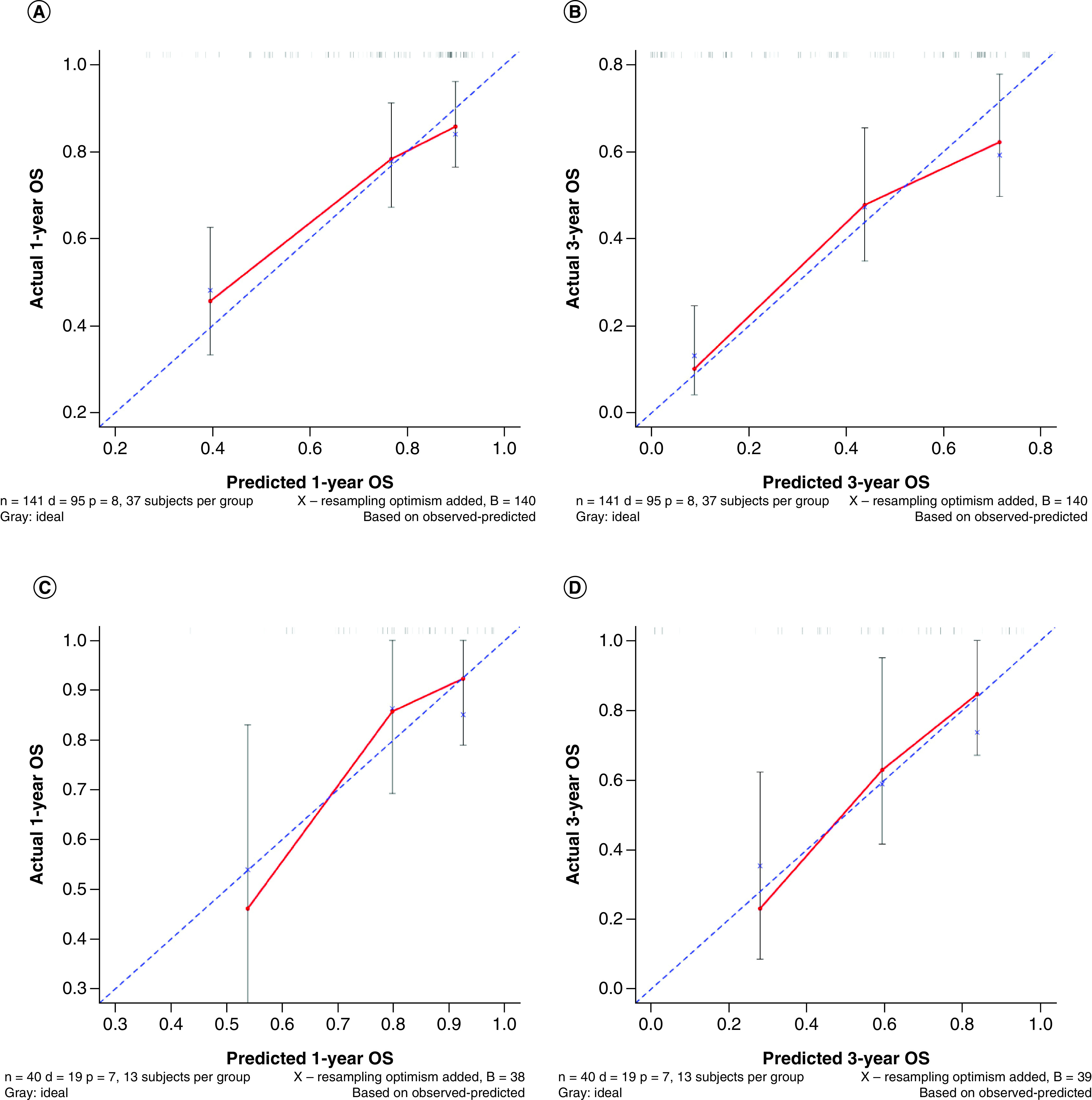
Calibration plot for the overall survival nomograms. **(A & B)** Calibration curves of nomogram to **(A)** 1-year overall survival and **(B)** 3-year overall survival in the training cohort. **(C & D)** Calibration curves of nomogram to **(C)** 1-year overall survival and **(D)** 3-year overall survival in the verification cohort. OS: Overall survival.

According to the results of the Cox regression analyses, a nomogram prognostic model was established to predict 1-year and 3-year survival probabilities. According to the nomogram, NLR <4.84 had the highest score (100 points), followed by T stage = 2 (89 points), age ≥67 years (48 points), BMI ≥21.13 (68 points), CEI ≥1729 (43 points), chemotherapy (34 points) and KPS score >80 (26 points) (Figure 1). The C-index of the nomogram prognosis model was 0.709 (95% CI: 0.679–0.739). In the calibration chart, the closer the predicted and actual results, the closer the calibration curve and diagonal (Figure 2A & B). The total score of each patient was calculated according to the nomogram and patients were divided into two groups according to the cutoff value: the low group (total score <222) and the high group (total score ≥222).

### Nomogram model validation

Forty-one patients from the verification cohort were used to verify the nomogram model. According to the nomogram model, the total score of each patient in the verification cohort was calculated and risk stratification was performed. Seventeen cases were included in the low group (total score <222), and 23 cases were included in the high group (total score ≥222). The C-index of the verification cohort was calculated to be 0.720 (95% CI: 0.658–0.782), indicating that the model had a good predictive ability, and a calibration chart was drawn (Figure 2C & D).

### Comparison of the nomogram prognostic model with independent prognostic factors

**Figure 3.**
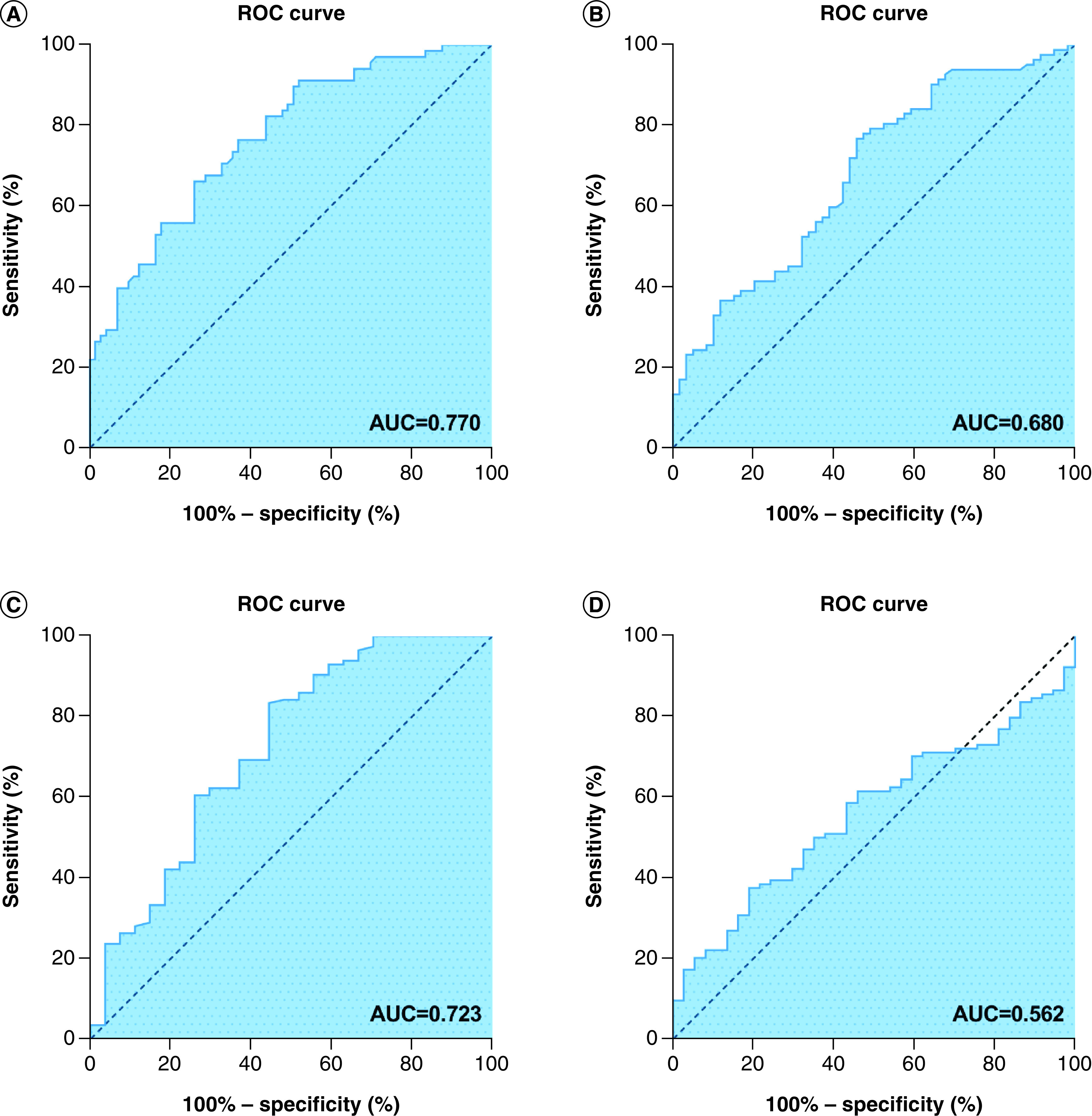
Receiver operating characteristic curve analysis of nomogram model, BMI, cervical esophageal carcinoma index and neutrophil to lymphocyte ratio. **(A)** Receiver operating characteristic (ROC) curve analysis of the nomogram model for overall survival (OS) in patients with esophageal squamous cell carcinoma (ESCC) (AUC = 0.731). **(B)** ROC curve analysis of the BMI for OS in patients with ESCC (0.598). **(C)** ROC curve analysis of the neutrophil to lymphocyte ratio for OS in patients with ESCC (0.506). **(D)** ROC curve analysis of the cervical esophageal carcinoma index for OS in patients with ESCC (0.514). AUC: Area under the curve.

The area under the receiver operating characteristic curve was used to compare the nomogram prognostic model with each independent prognostic factor. The results showed that the AUC of the nomogram prognostic model was 0.770, the AUC of the T stage was 0.659, the AUC of age was 0.600, the AUC for chemotherapy was 0.706, the AUC for the KPS score was 0.523, the AUC for BMI was 0.680, the AUC for NLR was 0.723 and the AUC for CEI was 0.562. Therefore the predictive power of the nomogram prognostic model was better than that of the independent prognostic factors (Figure 3).

### Relationship between prognostic score group & prognosis

**Figure 4.**
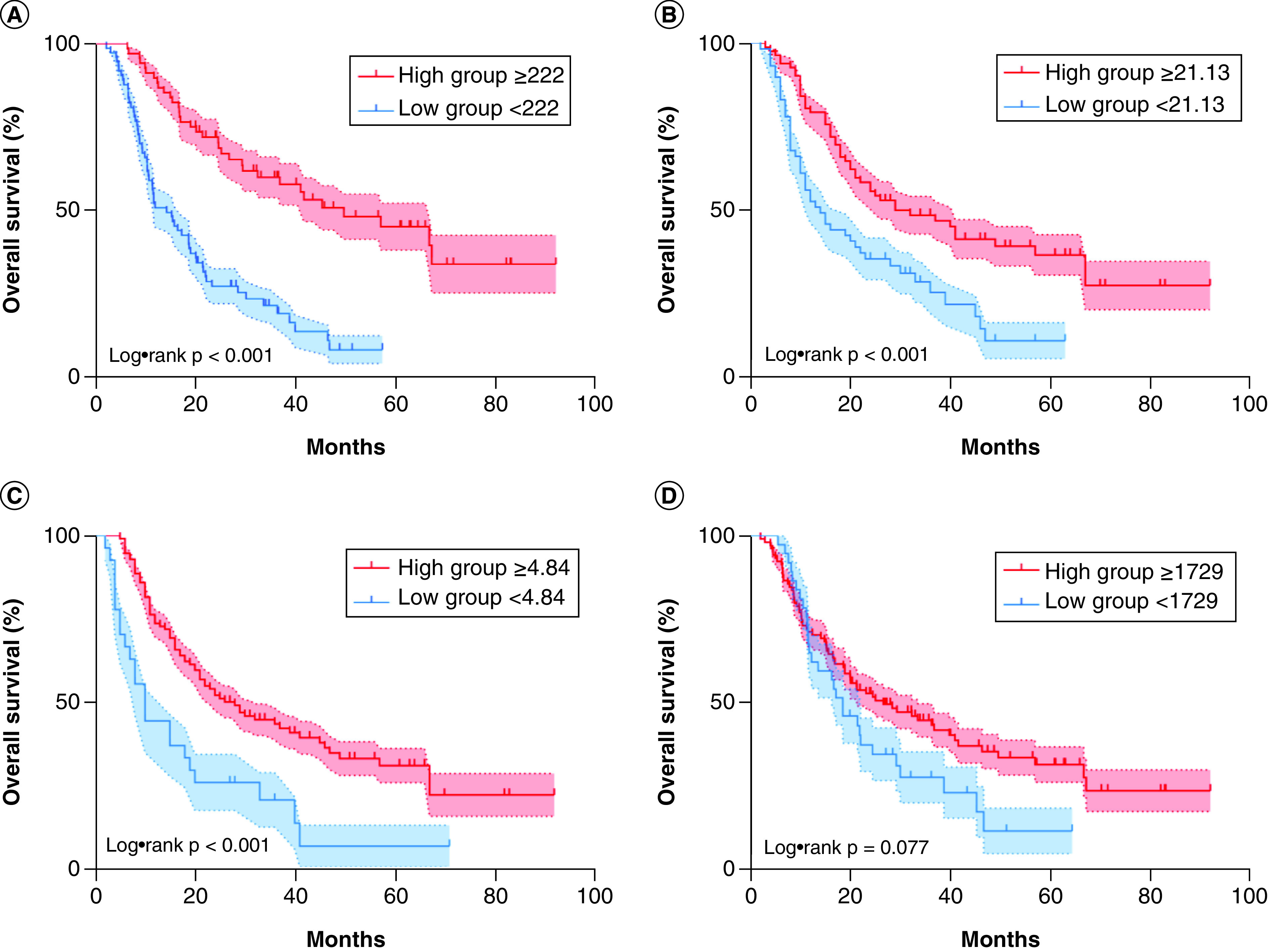
Overall survival of included patients stratified according to the before-treatment nomogram model, BMI, cervical esophageal carcinoma index and neutrophil to lymphocyte ratio cutoff values. The Kaplan–Meier curves and p-value represents the relationship between **(A)** overall survival (OS) and nomogram model (p < 0.001), **(B)** OS and BMI (p < 0.001), **(C)** OS and neutrophil to lymphocyte ratio (p < 0.001) and **(D)** OS and cervical esophageal carcinoma index (p = 0.077).

The Kaplan–Meier survival analysis method was used to draw the survival curve of the total score group. The results showed that the higher the total score, the longer the OS. The median OS of the training group in the low group (<222) was 14.4 months, while the median OS in the high group (≥222) was 49.9 months; the difference was statistically significant (p < 0.001 [Figure 4A]). The median OS of the high BMI group (≥21.13) was 29.6 months, while the median OS of the low BMI group (<21.13) was 14.4 months. This difference was also statistically significant (p < 0.001 [Figure 4B]). The median OS of the high CEI group (≥1729) was 26.9 months, while the median OS of the low CEI group (<1729) was 18.8 months; however, this difference was not statistically significant (p = 0.077 [Figure 4C]). The median OS of the low NLR group (<4.84) was 28.7 months, while the median OS of the high NLR group (≥4.84) was 10.5 months; this difference was also not statistically significant (p < 0.001 [Figure 4D]).

## Discussion

ESCC patients have a poor prognosis, and a common research goal of scholars is to identify a prognostic model with better predictive efficacy. Given the individual differences among ESCC patients, the selection of a suitable treatment plan is essential to improving their survival prognosis. To the best of our knowledge, there are few studies on the development and validation of nomograms for the prognosis of ESCC patients based on multiple blood prediction models. We have included a total of 18 factors. Using single factor analysis, 13 factors – age, KPS, tumor length, T stage, N stage, stage, chemotherapy, BMI, PNI, PLR, SII, CEI and NLR – were found to be related to OS. Multifactor analysis was also conducted and revealed that age, KPS, T stage, chemotherapy, BMI, CEI and NLR were independent prognostic factors in ESCC. We inputted them into the nomogram model, assigned scores to each factor, obtained the total score (39–314) by adding the scores, and finally calculated the function conversion relationship between the total score and the probability of the occurrence of the ending event. The C-index was used to verify the accuracy of the model. When the C-index is >0.7, we believe that the accuracy of the prognosis prediction is high [12]. The internally verified C-index of this model was 0.709 (95% CI: 0.679–0.739), and the externally verified C-index was 0.720 (95% CI: 0.658–0.782), indicating that the model can be used to predict the prognosis of ESCC patients.

Previous studies have shown that a high BMI is associated with an increased risk of various cancers, including lung cancer, colon cancer and breast cancer [13–15]. However, studies by Takenaka *et al.* showed that underweight patients with head and neck tumors have the lowest 5-year survival rate [16]. Our research indicates that a high BMI before treatment was an independent prognostic factor for ESCC patients (HR: 0.574; 95% CI: 0.376–0.878, p = 0.010). This is likely due to the special anatomical and physiological functions of the esophagus, and the incidence of malnutrition in patients with esophageal cancer is high. Study has reported that 60%~85% of patients with esophageal cancer have varying degrees of malnutrition [17]. Malnutrition will increase the adverse effects of RT, extend the length of hospital stays, increase RT positioning errors, affect the accuracy of RT, reduce the sensitivity of RT and reduce the short- and long-term efficacy of treatment [18,19].

Han *et al.* [20] reported a retrospective analysis of 206 patients with ESCC after esophagectomy and found that a high PNI had a positive effect on OS, but that PNI was not an independent prognostic factor, which is consistent with our study. However, there are some controversies about PNI in esophageal cancer research. In a retrospective analysis of 106 cervical ESCCs, Dai *et al.* [7] reported that compared with the low PNI group, the high PNI group showed a higher OS, and found that PNI was an independent prognostic factor. The reason for the difference may be that the influencing factors included in each study are different, or that the difference in the selection of the best cutoff value may cause different results when performing multifactor analyses.

Inflammation plays an important role in the development of cancer. Cancer patients with low lymphocyte counts in the blood are in an immunosuppressed state, and this can lead to poor prognosis [21]. In addition, studies have shown that platelets play key roles in inflammation and can promote the development of colorectal cancer [22]. Consistent with this idea, the use of anti-platelet production drugs can suppress the recruitment of immunosuppressive myeloid-derived suppressor cells, thereby inhibiting the occurrence of tumors [23]. Similarly, the neutrophils of cancer patients play an important role in cancer. In the blood analysis of many patients with advanced cancer, the number of neutrophils is significantly increased and the prognosis is poor [24]. Duan *et al.* showed that high NLR values before surgery are associated with tumor recurrence and poor clinical prognosis and are independent prognostic factors [10]. This is consistent with the results of this study. The low NLR value (<4.84) was associated with the highest score (100) when assigned through the nomogram, suggesting that it has an important predictive role in the construction of a new model. Interestingly, the results of the multifactor analysis found that NLR was significantly better than PLR in predicting OS, which is consistent with the results of previous studies [25].

Compared with traditional statistical models, the nomogram model is intuitive and easy to understand. It can predict the clinical outcome of different individuals and other characteristics and can also be used to guide treatment strategies. In addition, Deng et al. [26] demonstrated that the nomogram shows a good prognostic effect in the training and verification groups (5-year OS AUC: 0.685 and 0.744, respectively), which is similar to our research results. The nomogram we constructed is in the training and verification queue, and shows the prognostic results (total OS AUC: 0.770 and 0.744, respectively). We also applied the C-index to verify the accuracy of the model. The internally verified C-index of this model is 0.709 (95% CI: 0.679–0.739), and the externally verified C-index is 0.720 (95% CI: 0.658–0.782), indicating that the model can be used to predict the prognosis of ESCC.

This study has several limitations. First, this is a single-center, small-sample, retrospective study, and treatment bias is inevitable; thus large-scale multi-center, prospective studies are needed to verify the accuracy and practicability of the prognostic model. Second, the occurrence of esophageal cancer is related to certain geographical factors, living and eating habits, genetics and other factors; these factors need to be further included in order to have sufficient curative effect and absolute value for clinical application. Finally, because the model is based on multiple clinical records and blood test indicators, it may be too cumbersome and require further optimization.

## Conclusion

The ESCC prognosis model established in this study was confirmed by verification that the model could better distinguish patient risks and predict patient OS and can lay a foundation for prospective research. The nomogram can be used as a reliable tool for clinical decision-making, but a larger sample is needed to verify whether the model can be more widely used in clinical practice.

**Summary points**

- The prognostic models constructed by nomogram produce more effective predictions.
- Due to the special anatomical location and physiological function of the esophagus, nutritional status is correlated with the prognosis of esophageal squamous cell carcinoma (ESCC) patients.
- Concurrent chemoradiotherapy could improve the prognosis of ESCC patients.
- The immune status of ESCC patients was positively correlated with prognosis.
- The nomo map is simple and intuitive to predict the prognosis of ESCC patients, and an online evaluation tool can be developed in the future.
